# A Dual-Process Model of Subjective Aging: The Role of Temporal and Social Comparisons

**DOI:** 10.1093/geroni/igaf122.219

**Published:** 2025-12-31

**Authors:** David Weiss, M Clara de Paula Couto, Klaus Rothermund

**Affiliations:** University of Halle, Halle (Saale), Sachsen-Anhalt, Germany; Friedrich-Schiller University Jena, Jena, Thuringen, Germany; Friedrich-Schiller University Jena, Jena, Thuringen, Germany

## Abstract

We propose and test a dual process model of subjective aging illustrating how social and temporal comparison processes shape subjective aging in opposite ways in the second half of life. First, an information-processing pathway primarily involves temporal comparisons, including reflective processing that leads to assimilation. Second, a motivational pathway involves social comparisons, including evaluative processing that results in contrast. We predicted and found across two studies – a 10-year longitudinal (Study 1, N = 2,425, 39-93 years; 55.5% women) and an experimental study (Study 2, N = 160, 50-75 years, 58% women) - that social comparisons (“me vs. them”) result in more positive self-perceptions of aging and feeling relatively younger, whereas temporal comparisons (“me vs. past/future me”) lead to more negative self-perceptions of aging and feeling relatively older. We discuss how these insights can enhance our understanding of the cognitive and motivational processes that shape subjective aging and can inform interventions and policies aimed at fostering positive self-perceptions of aging and promoting healthy aging.

